# Strategies to Mitigate Age-Related Bias in Machine Learning: Scoping Review

**DOI:** 10.2196/53564

**Published:** 2024-03-22

**Authors:** Charlene Chu, Simon Donato-Woodger, Shehroz S Khan, Tianyu Shi, Kathleen Leslie, Samira Abbasgholizadeh-Rahimi, Rune Nyrup, Amanda Grenier

**Affiliations:** 1 Lawrence Bloomberg Faculty of Nursing University of Toronto Toronto, ON Canada; 2 Knowledge, Innovation, Talent, Everywhere (KITE) Toronto Rehabilitation Institute University Health Network Toronto, ON Canada; 3 Institute for Life Course and Aging Faculty of Social Work University of Toronto Toronto, ON Canada; 4 Rehabilitation Sciences Institute University of Toronto Toronto, ON Canada; 5 Institute of Biomedical Engineering University of Toronto Toronto, ON Canada; 6 Department of Civil Engineering University of Toronto Toronto, ON Canada; 7 Faculty of Health Disciplines Athabasca University Athabasca, AB Canada; 8 Department of Family Medicine McGill University Montreal, QC Canada; 9 Centre for Science Studies Department of Mathematics Aarhus University Aarhus Denmark; 10 Factor-Inwentash Faculty of Social Work University of Toronto and Baycrest Hospital Toronto, ON Canada

**Keywords:** age, ageing, ageism, aging, algorithm, algorithmic bias, artificial intelligence, bias, digital ageism, elder, elderly, geriatric, gerontology, machine learning, older adult, older people, older person, review methodology, review methods, scoping, search, searching, synthesis

## Abstract

**Background:**

Research suggests that digital ageism, that is, age-related bias, is present in the development and deployment of machine learning (ML) models. Despite the recognition of the importance of this problem, there is a lack of research that specifically examines the strategies used to mitigate age-related bias in ML models and the effectiveness of these strategies.

**Objective:**

To address this gap, we conducted a scoping review of mitigation strategies to reduce age-related bias in ML.

**Methods:**

We followed a scoping review methodology framework developed by Arksey and O’Malley. The search was developed in conjunction with an information specialist and conducted in 6 electronic databases (IEEE Xplore, Scopus, Web of Science, CINAHL, EMBASE, and the ACM digital library), as well as 2 additional gray literature databases (OpenGrey and Grey Literature Report).

**Results:**

We identified 8 publications that attempted to mitigate age-related bias in ML approaches. Age-related bias was introduced primarily due to a lack of representation of older adults in the data. Efforts to mitigate bias were categorized into one of three approaches: (1) creating a more balanced data set, (2) augmenting and supplementing their data, and (3) modifying the algorithm directly to achieve a more balanced result.

**Conclusions:**

Identifying and mitigating related biases in ML models is critical to fostering fairness, equity, inclusion, and social benefits. Our analysis underscores the ongoing need for rigorous research and the development of effective mitigation approaches to address digital ageism, ensuring that ML systems are used in a way that upholds the interests of all individuals.

**Trial Registration:**

Open Science Framework AMG5P; https://osf.io/amg5p

## Introduction

The rapid progress of machine learning (ML) has revolutionized health care decision-making, medical diagnosis, and other domains [1]. However, as the influence of ML systems expands, so do concerns regarding potential fairness issues that may arise from ML systems encoding human biases [2]. As an example, population health management systems have been found to underestimate the health risks facing Black patients, who are typically underrepresented in health care data due to systemic challenges accessing health care [3]. Concurrent with the rise of ML, there has also been a growing demand for efforts to improve the fairness of ML systems by better representing systemically disadvantaged groups in their data [4,5], such as gender and ethnic minority individuals [6-8]. To better understand the scope of this problem, frameworks to classify the various forms of bias present in ML have been developed. Our previous work used the framework developed by Mehrabi et al [5], which classified numerous types of bias according to the characteristics of each bias as well as where it would be introduced into an ML system in the cycle of providing training data (data to algorithm), the ML model interacting with the public (algorithm to user), and the public’s data being used for future testing (user to data). In our earlier investigation, we delineated 9 distinct categories of ML bias that could provide avenues for age-related bias to affect ML systems [9], using the conceptual framework by Mehrabi et al [5]. In this investigation, the prevalent forms of bias from their framework were: (1) representation bias, which emerges when the data set used for training the ML model inadequately reflects the diversity of the user population compromising the performance for specific demographic groups; (2) evaluation bias, which can occur when the model is tested with unrepresentative data and inappropriate evaluation benchmarks are used; (3) aggregation bias, in which distinct demographics within a larger sample are categorized in a way that makes their unique characteristics indistinguishable; (4) algorithmic bias, where the algorithm itself is the origin of the bias leading to distorted outcomes; and (5) measurement bias, which arises from how certain features are selected, measured, and used. When data are measured or gathered using improper tools or techniques, the resulting evaluation of the data by an ML model does not reflect the relevant variables within the data [5,9]

Digital ageism is a form of ageism perpetuated through the development, use, and deployment of technology and ML models [10,11]. Recently, the World Health Organization released a brief report about age-related bias in ML models and raised critical questions about the equitable treatment of older people across various sectors [12]. The rising concerns about digital ageism highlight the pressing need for further research and policy interventions to address the potential biases and discriminatory practices that may affect older adults in the digital era [13-15]. Recent studies have demonstrated instances of digital ageism, emphasizing the urgency of designing and deploying technologically inclusive solutions to ensure equitable treatment and opportunities for individuals of all ages [16]. The exclusion of older adults from the development of digital technologies has been previously researched [15,17,18] and can manifest in many ways. Older adults may not be adequately represented in training and testing data for ML models, resulting in models with reduced accuracy for older adult data and being vulnerable to multiple intersecting disadvantages [19]. For example, older adults who live in long-term care homes may have limited access to the internet and may be excluded from technological advances [20]. Data may also aggregate older adults into arbitrary age blocks, replicating problematic assumptions that link functional decline with age and failing to represent the diversity of the older adult population [10]. The marketing strategies for these ML systems often highlight their use in health care, reinforcing the idea of aging as a period of physical and mental decline [21]. As ML models and technologies become inextricably part of accessing opportunities and services, older adults’ risk of being left behind by a growing digital divide increases [22]. This is particularly alarming considering that the older population represents the fastest-growing demographic worldwide [23].

The topic of digital ageism is gaining prominence in scholarly discussions, leading researchers to investigate these phenomena from various perspectives [9,19,24,25]. Previous investigations have focused on developing conceptual frameworks to comprehend and define the nature and implications of digital ageism [13]. Previous reviews of facial image data sets have also found that older adults, particularly older adults aged 85 years or older, are underrepresented in a majority of data sets [18]. While this research has been foundational in identifying and characterizing these biases, there is now a critical need to focus on the mitigation strategies that can address age-related bias in ML models. The purpose of this scoping review is to advance this crucial discussion by shedding light on the mitigation strategies currently being used to address age-related bias in ML models. By bridging the gap between theory and practice, this research aims to pave the way for meaningful and impactful interventions that can rectify biases and promote inclusivity in the digital age. Our research focuses on two main questions: (1) Which mitigation strategies have been used to address age-related bias in artificial intelligence, and how successful were these strategies? and (2) Specifically, what types of biases were targeted and mitigated during these efforts?

## Methods

### Overview

This review is part of a larger scoping review about digital ageism that follows a 6-stage methodology framework developed by Arksey and O’Malley [26] and further refined by Levac et al [27]. A scoping review was appropriate to explore the study’s aims of summarizing the available evidence, identifying gaps, and establishing future directions regarding mitigation strategies. As the breadth and depth of the literature are unclear, a wide and interdisciplinary approach was used [10]. The description of the review was published elsewhere [28] and registered in the Open Science Framework database [29]. This review also followed the PRISMA-ScR (Preferred Reporting for Items for Systematic Reviews and Meta-Analyses Extension for Scoping Reviews) format [30].

### Information Sources and Search

An information specialist helped develop the search strategy in Scopus, which was then translated into 5 other databases (Web of Science, CINAHL, EMBASE, IEEE Xplore, and the ACM digital library). The search strategy included the terms “machine learning,” “artificial intelligence,” “algorithms,” “neural networks,” “deep learning,” “algorithmic bias,” “biased,” “discrimination,” “ageism,” “age,” and “older people.”

### Eligibility Criteria

Articles were included if they were published in English and focused on “artificial intelligence” in the context of algorithms that make predictions and classifications about data; “bias”; and age-related terms such as “aging,” “older,” and “demographic.” As the term “artificial intelligence” is over 50 years old [31], the search strategy was also not restricted by publication date, and databases were searched from inception. Papers were excluded if they included nonhuman topics. Theses, conference abstracts, dissertations, nonpeer-reviewed conference proceedings, books and book chapters, perspectives, editorials, and editorial letters were also excluded.

### Selection of Sources of Evidence and Charting the Data

**Figure 1:**
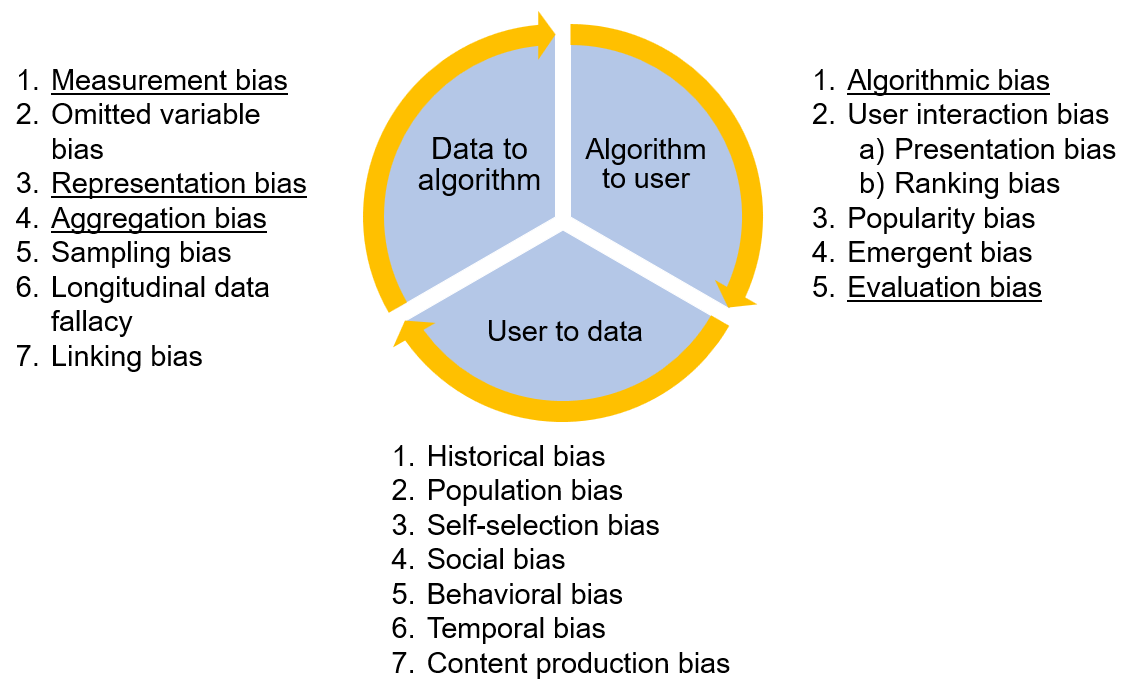
The framework by Mehrabi et al for bias in machine learning. The specific biases discussed in this review are underlined.

The academic literature search was completed in January 2022. All citations were uploaded to Covidence (Veritas Health Innovation), a systematic review software, and duplicates were removed. The titles and abstracts of all articles were screened by 2 independent reviewers according to the eligibility criteria. Once the abstract screening was complete, the full text of each article was reviewed by 2 independent reviewers to judge the article’s relevance to the research questions. Data extraction included the manuscript information (title, authors, year, and location), study design, type of ML model and purpose, database used, type of data, presence of age-related bias, mitigation strategy used, and effectiveness of the strategy if reported. The framework by Mehrabi et al [5] (Figure 1 [5]), which identified different sources of biases that can affect ML according to the data-to-algorithm (data), algorithm-to-user (modeling), and user-to-data (deployment) interaction loops, was used in this review to identify the different types of bias in the included studies [5]. A total of 5 of the 19 different types of biases in the framework by Mehrabi et al [5] (Figure 1) were included in the extraction table, including representation and evaluation bias, aggregation bias, measurement bias, and algorithmic bias.

Studies were selected if they acknowledged the presence of any bias against older adults in either their data or results, and the researchers then took any action to correct that bias, regardless of its effectiveness. For example, publications were selected based on whether authors attempted to enhance the performance of their model on older demographics, regardless of the success of their efforts. Biweekly meetings were held to discuss the progress of the charting process. Disagreements were resolved through discussion or by having the first author (CC) act as a third reviewer. The extracted information was converted into table format, which allowed the authors to develop a narrative description according to the type of mitigation strategy (Table S1 in Multimedia Appendix 1 [32-42]). The team conducted additional analysis of the databases in the included studies to identify data disparities among older adults and provide further directions for future studies in the field of digital ageism. The results of the literature search are reported in tables. One of the challenges involved in assessing the inclusion of older adults involves defining the age at which someone is considered “old.” While the commonly accepted age for legal recognition as a “senior citizen,” based on general eligibility for a public pension, is 65 years [43], the data sets and articles we reviewed grouped older adults into a much wider range of age categories (Table S2 in Multimedia Appendix 1), starting with adults aged 50 or older. When we refer to “older adults” throughout this paper, we are referring to either (1) the participants in the data set aged 60 years or older, or else (2) the oldest age category found in the data set or publication being discussed.

### Ethical Considerations

Ethics approval was obtained from the (University of Toronto) Research Ethics Board (REB #40095) for a larger study on the same topic. This study does not contain any studies with human participants performed by any of the authors.

## Results

### Overview

**Figure 2:**
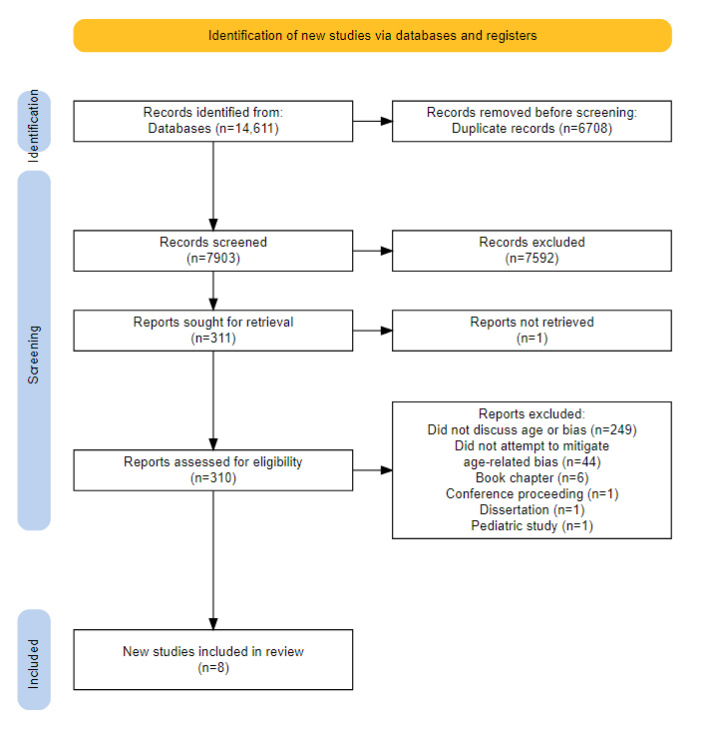
PRISMA (Preferred Reporting Items for Systematic Reviews and Meta-Analyses) flowchart of the literature review.

From our search, 14,611 academic publications were identified. After removing duplicates, we screened the abstracts of the remaining 7903 publications. During the abstract screening process, we excluded 7592 publications. Subsequently, we conducted a full-text screening of 310 articles, ultimately including 8 academic publications in this review (Figure 2).

### Types of Ageist Bias in the Selected Publications, per the Framework by Mehrabi et al

After reviewing the full text of each of the 8 publications, 5 types of bias found in the framework by Mehrabi et al [5] (Figure 1) were identified. Representation bias occurs when the data set underrepresents or misrepresents specific demographics within the overall population, resulting in a nonrepresentative data set [5]. Evaluation bias is similar to representation bias: it occurs when inappropriate evaluation benchmark data are selected to assess ML models [5]. In this review, this amounted to using the same underrepresentative data sets for training as well as testing the model, which was found to be the case in 7 publications [32-38]. Aggregation bias occurs when conclusions are drawn based on observations about a larger group, overriding unique characteristics about a smaller demographic within that group [5]. As a result, the data set fails to account for the unique characteristics of more specific demographics within the overall data set. Aggregation bias was found in 2 publications in this review [34,35]. Algorithmic bias occurs when the bias is generated at the level of the algorithm’s calculation itself rather than being a by-product of biased data or measurement tools being provided to that algorithm. Algorithmic bias was found in 2 papers in this review [36,39]. Measurement bias occurs when the data being processed by the algorithm fail to represent the variable of interest accurately. It can often arise from the methods used to collect or measure the data or respective variables. Measurement bias was demonstrated in 1 publication in this review [35]. A complete list of each type of bias found in each publication, along with a rationale, can be found in Table S1 in Multimedia Appendix 1.

### Data Sets Used in the Included Studies

Table S2 in Multimedia Appendix 1 presents the demographic breakdown of the data based on age to determine the extent of underrepresentation of older adults in common data sets. Overall, there was a large data disparity between the data of older adults compared with younger individuals in all the databases in this review. In the FG-Net data set, the oldest age group was “aged 61 years old or older.” In this age group, the FG-Net data set only had 7 images (0.7%) [40]. In the MORPH Academic data set, there were only 3933 (7.1%) images of individuals in the “aged between 50 and 77 years” category [44], and in the MORPH Longitudinal data set, only 5615 (1.4%) of the data set’s 402,055 images were from the “aged between 60 and 69 years” (5021 images) and “aged 70 years or older” (594 images) categories [45]. The CACD data set contains 163,446 images, divided into age groups of 10 years (0-10 years, 10-20 years, 20-30 years, 30-40 years, 40-50 years, 50-60 years, and 60 years or older). However, only 2912 (1.78%) of these images depict participants older than 60 years (the fifth figure in Georgopoulos et al [34]). Grouping all older adults into one 60+ demographic category also raises the risk for aggregation bias [5].

The APPA-Real data set contains 7591 images (from public internet repositories), aged between 10 and 95 years. When we combine the APPA-Real data set’s 4 oldest age groups: between 60 and 70 years (254 images), between 70 and 80 years (111 images), between 80 and 90 years (68 images), and between 90 and 95 years (13 images), the combined total of 446 images accounts for just 6% of the entire data set [41]. Lastly, the 100 Celebrities data set created by Jung et al [35] has a smaller-sized balanced data set to offset the imbalances in the IMDB-Wiki and Twitter (subsequently rebranded as X) Profile data sets also used in the same study. Participants were divided into 3 age groups: between 14 and 34 years (33 images), between 35 and 55 years (34 images), and 55 years or older (33 images), with the “55 years or older” age group having equal representation with the other demographics, although grouping all older adults into 1 category of aged 55 years or older increases the likelihood of aggregation bias [5].

For the other data sets present in this review (11,000 Hands, ABIDE, CoRR, DLBS, NKI Rockland, Pilots Parliament Benchmark, and a data set comprised of Twitter Profile data), the exact data for the age demographics in each data set was not readily available (Table S2 in Multimedia Appendix 1).

### Bias Mitigation Strategies

We found 8 studies that attempted to mitigate bias against older adults. The studies were all related to “computer vision” systems, systems that rely on an ML model’s processing and interpretation of images, although with varying aims: 1 study focused on hand images [32], another study focused on radiological scan interpretation [36], and 6 others focused on facial images [33-35,37-39]. A complete list of the papers, the data sets that were used, and the strategies used to mitigate bias and their outcomes can be found in Table S1 in Multimedia Appendix 1. We identified 3 broad categories of bias mitigation strategies: data set balancing, data set augmentation, and algorithm alterations. This section will provide a comprehensive overview of each bias mitigation strategy and its effectiveness [34,36].

#### Data Set Balancing

Data set balancing involves the practice of ensuring balance within the data sets used for training and testing ML models [5]. This strategy aims to address representation bias due to the imbalance in the representation of older adults and subsequent evaluation bias against different age groups in the data set, which can lead to biased predictions and unfair outcomes [5]. By ensuring a more balanced distribution of samples across age groups, the model can learn from a more diverse and representative set of examples, reducing the potential for age-related bias. A total of 4 papers used data set balancing techniques to achieve an equitable distribution of data across different classes or categories within the data sets, such as altering their data set or creating a new data set (as was the case for Jung et al [35]) that would balance their previously unrepresentative data.

For their study to demonstrate the effect of bias on older adults with dementia on facial expression analysis models, Taati et al [39] developed a data set of test participants comprised entirely of older adults for their study. This data set was comprised of images of 86 older adults (aged 65 years or older), of whom 42 were affected by dementia and 44 were cognitively healthy, creating a balanced data set with the target population [39]. Frontal and profile photos of each participant’s face were taken at baseline, and a physiotherapist guided each participant through a series of exercises to identify painful positions. Images were then annotated manually according to the facial action coding system (FACS) and the “Pain Assessment Checklist for Seniors with Limited Ability to Communicate-II” (PACSLAC-II) pain scales. This method helped avoid potential representation biases that could arise from an imbalanced representation of different cognitive states. Taati et al [39] also used a fine-tuning method, in which they pretrained their models with images of cognitively healthy older adults and then fine-tuned their models with images of cognitively impaired older adults. They found that when this strategy was tested on active appearance models (AAMs), the number of images in the fine-tuning data set with a normalized root-mean square error (NRMSE) of <5% improved from 87% to 91% accuracy. However, when fine-tuned with the same strategy, facial alignment networks (FANs) performance remained around 90% (for an NRMSE threshold of 5%). When the NRMSE threshold was lowered to 4%, the performance disparity became even more significant: AAMs and FANs both started between 65% to 70%, but the number of fine-tuning images with an NRMSE of <4% continually increased into the 75% to 80% range when the AAM was fine-tuned, while FANs did not see any increase (second and third figures in Taati et al [39]). The gaps in performance between AAMs and FANs indicate that the bias present in these results is at least partially algorithmic in nature, as both models were tested using the same strategy but only the AAMs showed any improvement [5].

In another paper, Zou et al [38] tested a model intended for cost-sensitive facial age estimation using the FG-Net and MORPH data sets, along with an image database of 14,238 images taken from Wikipedia Commons. They modified the FG-Net data set by adding images from their Wikipedia data set to balance it for age, particularly for the groups aged between 40 and 49 years and between 50 and 59 years. Doing this corrected the representation bias in the FG-Net database, 87% of which consists of participants aged 30 years or younger [38]. This mitigation strategy effectively resulted in a significant reduction in mean absolute error (MAE) for those age groups, along with a smaller reduction in MAE for the other age groups in their test. The balanced data set using the cost-sensitive function showed the lowest MAE in age predictions (MAE 8.25, SD 0.03) versus the cost-insensitive data set (MAE 9.31, SD 0.4) and an unmodified data set (MAE 8.6). This approach effectively reduced the MAE for the groups aged between 40 and 49 years and between 50 and 59 years, so it was similar to the younger groups aged between 20 and 29 years and between 30 and 39 years. However, the representation of the groups aged between 60 and 69 years and 70 and 79 years appear to have been largely unchanged (fifth figure in Zou et al [38]), with every other age category receiving a substantial number of additional images except for the 2 oldest, and as a result, the MAE for these groups seems to be substantially higher, even using the cost-sensitive model (eg, for the group aged between 60 and 69 years, the MAE was 17, and for the group aged between 70 and 79 years, the MAE was 30), while the younger age groups did not have an MAE above 10 [38].

Jung et al [35] worked with several databases on facial recognition, including the IMDB-Wiki data set, a data set composed of Twitter profile images, and the 100 Celebrities data set. Jung et al [35] created the 100 Celebrities data set after noticing that the other data sets were imbalanced for age and ethnicity. Celebrity images were selected due to the wide availability of high-quality images of celebrities from a wide range of angles and the simplicity of establishing the participant’s true age when the photo was taken (celebrity birthdates are easily determined). Creating this data set would help mitigate the representation and evaluation bias found in the IMDB-Wiki data set, although using those data sets without balancing them would still expose the outcomes to those biases [5]. Age detection by the Face++, IBM Bluemix Visual Recognition, AWS Rekognition, and Microsoft Azure Face API detection systems found that on the balanced 100 Celebrities data set, age was underestimated by 15.2 years. The highest accuracy of all the models trained on the 100 Celebrities data set was the IBM model, at 53% (although the Microsoft model achieved 66% accuracy when trained on the Twitter-Age data set). However, it is worth noting that the 100 Celebrities data set is comprised of celebrities who fall under entertainment-industry beauty standards, which may explain the large variance. While the creation of a balanced data set is a step toward mitigating biases, the fact that the age detection models still exhibited significant inaccuracies in this study raises questions about the overall effectiveness of the strategy with this specific data set. By grouping all older adults into a single category (aged 55 years or older), the 100 Celebrities data set may also increase the likelihood of aggregation bias, as mentioned in the previous section.

Finally, Liang et al [36] attempted to balance their combined data set that originally contained magnetic resonance imaging (MRI) scans of the brain from 2026 participants. These were comprised of samples from the ABIDE, CoRR, DBLS, and NKI Rockland data sets that had a higher number of participants aged 40 years or younger. To help balance representation bias within this sample, the authors resampled the full data set in 5-year age intervals to address overrepresentation and underrepresentation of age groups. They duplicated samples from overrepresented age groups to match the number of samples in underrepresented age groups (ie, participants aged 75 years or younger), resulting in a more balanced distribution of age groups within the new data set (n=782), reducing the bias toward certain age ranges, and ensuring a more representative representation of the population [26]. However, the researchers noted that a significant bias persisted even after training their model on their more balanced data set. Testing their model on the imbalanced data set returned *r*=.91 and an MAE of 6.77 years, while the balanced data sample returned *r*=.91 and an MAE of 8.02 years. The correlation between the brain-age gap and chronological age remained the same (–0.52) for both the imbalanced full data set and the balanced data sample. This indicates that, despite accounting for representation bias by balancing the data set, the strategy alone did not achieve the desired reduction in bias. Moreover, the MAE increased from 6.7 years to 8 years; although the correlation remained the same, the higher MAE suggests a decrease in the accuracy of the model’s predictions when using the balanced data set. These findings suggest that additional strategies or factors may need to be considered to further mitigate bias and improve the accuracy of the model. It is possible that the resampling strategy, while addressing representation bias to some extent, may not have fully addressed other sources of bias present in the data set. Therefore, this strategy had limited effectiveness in addressing bias while aiming to improve accuracy.

#### Data Augmentation

Data augmentation strategies can mitigate bias related to ageism in ML models by enhancing the diversity and representativeness of the data set. This enables the model to learn from a wide range of age groups, reducing potential bias toward specific age categories. In 3 papers that applied data augmentation techniques, the actual images in their data set were modified without adding images from an external source. For example, Georgopoulos et al [34] applied digital age progression methods to the images in the data sets selected for their study, generating realistic images of what their participants may look like as they age but modified images of the participant. Smith and Ricanek [37] used random cropping and Gaussian tinting, and Abderrahmane et al [32] used an unspecified technique.

Using the MORPH, Cross-Age Celebrities data set (CACD), and FG-Net data sets, Georgopoulos et al [34] tested the ability of a generative adversarial network (GAN) to synthesize aging patterns realistically. They divided the participants’ images from each data set into 4 groups: those aged 30 years or younger, between 31 and 40 years, between 41 and 50 years, and 51 years or older. For each data set, their model would then take an image from these data sets and either age or de-age new images for each of the other 3 categories to which the participant did not belong, creating new images for each of the other 3 age groups and balancing the data set in the process. The approach was effective in creating a data set 4 times the size of the original data set, and their results were able to significantly improve both the data set diversity (measured using the Shannon D and Simpson H metrics) and overall balance (measured using the Shannon E and Simpson E metrics) of the data sets they studied, demonstrating superior performance over contemporary models (Conditional Adversarial Auto Encoders [CAAEs] and Identity-Preserved Conditional Generative Adversarial Networks [IPCGANs]). While CAAEs generated the most accurate images for the group aged between 31 and 40 years after being trained on the MORPH data set (MAE 1.18), and IPCGANs generated the most accurate images for the demographic aged between 31 and 40 years after being trained on the CACD data set (MAE 0.04), the novel method presented in the paper was most accurate for the groups aged between 41 and 50 years and 51 years or older, for both the MORPH and CACD (MAE of 1.21 and 1.69, and MAE of 1.33 and 1.04, respectively). The images generated by the novel method also produced the best scores on the Simpson and Shannon data diversity indices after augmenting the MORPH, CACD, and FG-Net databases. This approach effectively reduced the representation and evaluation bias against older adults in these data sets. In doing so, the researchers acknowledged the possibility of their model enabling researchers to overcome demographic bias in facial image data sets, the most popular of which heavily underrepresent older adults [34]. The method used could also have the impact of increasing aggregation bias, as all older adults were grouped into a single group (aged 51 years or older) [5].

Smith and Ricanek [37] studied age and gender prediction models using data sets taken from IMDB, Wikipedia, and the MORPH data sets, which underrepresent older adults and present representation and evaluation bias [37]. They sought to expand the robustness of their data sets by applying random data-augmentation policies, which are transformation techniques used to modify existing data. For instance, they used random cropping and Gaussian tinting techniques to increase the diversity of the images in the data set. After training and testing, they also composed a separate challenge data set, which applied the data-augmentation policies to images from categories that their model had difficulty identifying accurately, including female individuals, older adults, and individuals with darker skin. The MORPH data set lacks images of older adults, so adding additional images was effective in addressing representation and evaluation bias in the data set [5]. The data augmentation policies would be randomly applied to these images as they were loaded during training [37]. This method to augment the training data was effective, as the overall MAE fell from 4.62 to 4.21, with a final MAE of 4.13 for male individuals and 4.90 for female individuals. The overall gender prediction accuracy increased slightly, from 98.44% to 98.92%.

Abderrahmane et al [32] developed an algorithm for age prediction based on hand images. They acknowledged that their data set initially had a highly imbalanced age distribution, which could introduce bias in their model. To address this, they used data augmentation techniques to create a more balanced data set from the 11,000 Hands Database, which contained 11,000 images of hands from 190 participants, showing the dorsal and palmar aspects. The authors recognized that the data set was underrepresentative of older adults and appeared to use data augmentation to balance the data set. There were no specific details regarding the augmentation processes used, but the figures suggest that additional images were added to the data set to address the underrepresentation of certain age groups. The underrepresentation of older adults in the 11,000 Hands data set is noteworthy in light of the significant use of hand images to represent older adults (more details on this issue have been provided in the *Discussion* section).

#### Algorithmic Alteration

Papers were listed under algorithmic alteration if the researchers adjusted the calculations of their algorithm itself and applied statistical methods to their algorithms to reduce the bias in their outcomes [5]. We found that 2 papers had adjusted their algorithms to improve performance [33,36]. Liang et al [36] applied linear regression to correct for the bias produced by their model after balancing the data set, which proved unsuccessful. Noticing a bias in age prediction based on MRI scans, which resulted in less accurate predictions for older participants, they unsuccessfully attempted to balance their combined data set, as was previously discussed. After examining multiple possible additional sources of the bias, including noise within the data, heterogeneity of the data sets, and the use of specific ML models, they were able to correct the bias statistically with a linear regression, fitting a linear regression model for predicted age to the chronological age and sex that improved MAE. By ruling out the other sources of bias (such as representation and evaluation bias, which they corrected for by balancing their data set), they were able to determine that the algorithm itself was the source of the issue (algorithmic bias, per the framework by Mehrabi et al [5]). The study by Liang et al [36] is unique in that they attempted to correct for more than one type of bias: first working on representation bias, and then solving for algorithmic bias (biases introduced by the algorithm itself) after balancing their data set was unsuccessful, which was an overall effective approach.

In the second paper, Clapes et al [33] sought to correct the bias between estimated age and true age by dividing participants into smaller groups based on mutually exclusive image categories and recalculating the estimated age for each real age. This produced a fitted curve for the difference between estimated and real ages, which was then used to correct the bias between the estimated and real ages. Linear interpolation was used to correct bias for ages that had fewer examples. This effectively addressed the measurement bias in the study. Clapes et al [33] also added labels for expression, ethnicity, makeup, gender, and the age of the photograph itself to the APPA real database. The resultant model’s overall performance improved, reducing the MAE from 13.57 to 12.07. The reduced representation of older adults is mirrored by trends in the consistency between age predictions in Clapes et al [33], where the difference between real age and estimated age widens as age increases (panels A-F in the eighth figure in Clapes et al [33]), which they theorized was due to the decreasing representation of older age groups (ie, representation bias).

## Discussion

### Overview

Our review included 8 publications that used mitigation strategies to address age-related bias. To our knowledge, this is the first review to examine this topic. Our analysis revealed that age-related bias predominantly stemmed from the underrepresentation of older adults in the data sets used to build the models (representation bias). Notwithstanding the 100 Celebrities data set, the data sets in this study (for which data were available) contained only 0.05% to 7% of data representing older adults. Our first research question explored the variety of strategies used to address this bias and their effectiveness. Researchers used three approaches: (1) creating a smaller yet more balanced data sample from their existing data set (n=4) [35,36,38,39], (2) augmenting and supplementing the available data (n=3) [32,34,37], and (3) modifying the algorithm directly to account for bias specifically (n=2) [33,36]. There was heterogeneity in the outcome measures, so a meta-analysis was not possible. Our findings emphasize the multifaceted nature of bias in ML models and the strategies available to address it, as well as the critical imperative of identifying and mitigating age-related bias in ML models to ensure fairness and equity for older adults in society [32-39].

The effectiveness of mitigation strategies aimed at reducing age-related bias in ML models varied based on several factors, including the types of data used, the ML approaches used, and the specific purpose of the ML model. While the included papers covered a range of uses, including facial and age recognition and MRI brain-scan interpretation, it is crucial for researchers to recognize that the solutions that may apply to one type of model may not apply to others due to qualitative differences in the data each model depends on. While many researchers reported successful outcomes with their methods, some encountered challenges and limitations. Jung et al [35] conducted experiments with a more balanced data set but found that the accuracy of their model reached a peak of only 53%. This suggests that achieving complete mitigation of age-related bias may be difficult, even with an improved data set balance. Taati et al [39] explored a mitigation strategy that showed mixed results and discovered that adding images of older adults affected by dementia to the training data for models trained on cognitively healthy older adults improved landmark detection significantly, but this approach improved the performance of AAMs significantly more than FANs. This highlights how specific types of algorithms can affect outcomes and how that must be considered when selecting which models to use when attempting to reduce bias. In a follow-up study, Asgarian et al [40] also identified performance differences in models intended to identify facial landmarks between healthy older adults and older adults with dementia [46]. In another example, Liang et al [36] encountered challenges in their attempts to balance data set representation and ultimately settled on a linear regression alteration for their algorithm. Making algorithmic alterations appears to have been most effective at mitigating measurement bias, but this is an extremely small sample (n=2) [33,36]. Overall, these findings highlight the complexity and context-dependence of mitigating age-related bias in ML models. While some strategies showed promising results, achieving complete elimination of bias remains challenging, and alternative approaches may need to be explored. Researchers must consider how both the data and the models they are using may affect outcomes.

Our second research question explored the types of bias being mitigated. Our previous work has demonstrated that age-related bias is present across the ML life cycle [9]. Bias affects ML models at multiple levels, including the data that trains the models (data to algorithm), the models themselves (algorithm to user), and the people who rely on the models (user to data) [5]. Our results found that researchers who attempted to correct for bias primarily focused on representation bias (n=7), with algorithmic bias (n=2) and measurement bias (n=1) being far less common targets of mitigation efforts. Representation and evaluation bias and measurement bias were most commonly mitigated by data set balancing [35,36,38,39], but this method did not consistently achieve the desired reduction in bias [35,36], while algorithmic bias [33,36] was resolved by algorithmic alterations, to the satisfaction of their respective authors.

Moreover, we noted that 2 papers may have aggregation bias [34,35], which occurs when a demographic is grouped in a way that does not account for its heterogeneity [5]. Many data sets in this review grouped all older adults into arbitrarily large categories (eg, those who were aged 55 years or older and 60 years or older). For example, Georgopoulos et al [34] aggregated the oldest participants into a single category of those aged 50 years or older [34]. While this worked to address one type of bias (representation bias), it reinforced another type of bias (aggregation bias), which impacts older adults significantly and essentially erases older adults as a distinct group, given that the legal cutoff to be considered a senior citizen is usually 65 years old [5]. While the 100 Celebrities data set by Jung et al [35] offered older adults equal representation compared with other age groups, it also grouped all older adults into a single category (ie, aged 55 years or older). This point of discussion holds significance but is often not fully acknowledged when tackling bias in ML. It highlights that bias can manifest in various interconnected ways, and while addressing the most prominent and evident forms of bias remains crucial, it may not completely eradicate bias from a model. Much of the discussion of bias in ML focuses on representation bias of specific demographics (it was the most common type of bias in this review), but there are other forms of bias that may be more easily neglected (such as aggregation bias, which was not addressed in the included studies). Sometimes, efforts to mitigate bias did not reach the data set’s oldest demographics. Panel 5A from the fifth figure in Zou et al [38] shows that the authors only balanced the FG-Net data set, and while they balanced the representation for all other age groups, they did not significantly improve the representation of the groups aged between 60 and 69 years and 70 and 79 years, which remained underrepresented. There are also ageist contradictions that emerge when representation is examined, with the strongest example in these papers being how even the images of the hands of older people were underrepresented in the 11,000 Hands data set, despite the prominence of wrinkled, disembodied hands as a dominant social representation of older people in media and societal images [47]. Such imagery may have a dehumanizing effect by portraying older adults as a pair of hands rather than a face or a whole person, which could result in their apparent exclusion from a data set of images with similar subject matter being compiled for practical purposes [48].

An important implication is for developers to consider the practical significance or real-world impact of the mitigation strategy. For example, Clapes et al [33] reduced their MAE from 13.6 to 12.1, while Smith and Ricanek [37] reduced their overall MAE from 4.6 to 4.2, with a final MAE of 4.1 for male individuals and 4.9 for female individuals. While these are quantifiable improvements, it is also important to consider the extent of an improvement and whether it has practical meaning or relevance in decision-making processes.

To address age-related bias in ML, researchers and designers can take steps from research design, methodology, and technical perspectives to ensure that technology is accessible and inclusive. From a research design perspective, an alternative mitigation strategy for developers collecting their data sets is to provide a representative sample of participants. Jung et al [35] created an age prediction training data set using celebrity images from IMDB. The researchers intended to create balanced data sets for their target populations, which aimed to prevent representation and evaluation bias, reducing the potential for age-related biases in the analysis and evaluation of the data. Although the effectiveness of this specific approach is not explicitly stated, the use of balanced data sets likely played a role in mitigating potential biases stemming from an imbalanced representation of age groups. Consequently, their study results may be more reliable and less susceptible to age-related biases, contributing to a more unbiased understanding of age recognition algorithms (although they may still be affected by other varieties of bias, as was previously discussed). By emphasizing the importance of representative sampling and the creation of balanced data sets, researchers can minimize the risk of introducing age-related biases into artificial intelligence systems and foster more accurate and equitable results.

Lastly, promoting the consideration of age as a variable of interest could help draw the attention of researchers and developers to digital ageism and disparities within their data sets. Recognizing age as a crucial factor requires a multidisciplinary approach that involves educational institutions, industry leaders, and policy makers. Within educational curricula, emphasizing the significance of critical demographic parameters can sensitize future engineers and computer scientists to the diverse needs of populations, including older adults. Technical approaches that use robust statistical approaches can begin to minimize age-related bias in ML. Techniques such as stratification and oversampling can ensure adequate representation of older adults in training data sets, attenuating the risk of underperformance or misclassification for this demographic group. These techniques are particularly relevant where the intersections of social locations of age and known underrepresentations of disability, race, and sexual orientation in ML models are concerned for 3 reasons. First, many of these types of bias (racism, sexism, and ageism) have their roots in similar problems, such as underrepresentation [4,9]. For example, a 2022 study of 21 age-recognition systems found that artificial intelligence systems consistently identified age with less accuracy across all age, gender, and ethnic categories, which the authors speculated was due to older adults being underrepresented in training data [49]. Second, the methods used to balance age-related bias in these papers may also have applications for other types of bias. For example, Sixta et al [50] reported a combination of strategies to reduce bias, including balancing their data for underrepresented demographics using external data and then further augmenting the data with different lighting and image quality adjustments. Third, the discussion around these types of bias in ML is much broader than the focus on digital ageism, making them an effective tool for drawing attention to these issues and advancing work in this area [9]. In a comparison of commercial facial emotional recognition (FER) systems, Kim et al [51] found that performance did improve for all demographics from 2019 to 2020, but that FER systems still performed best on younger adults and most poorly on older adults. The “black box” nature of these commercial systems, in which the method’s underlying algorithm is inaccessible, makes it impossible for external reviewers to assess the source of these improvements from an algorithmic standpoint [51]. However, digital ageism is not limited to ML and is a broader issue that stems from societal biases in the design, development, and deployment of ML models [9,16]. The multivalent nature of age-related bias in ML requires solutions from multiple sectors of society, including the public, private developers, government, and academic research [24]. Future research should focus on developing a global consensus of priorities that can mobilize the multitude of players from these sectors to advance knowledge about age-related bias and best practices to address this bias.

### Limitations

One limitation of this study is the exclusion of publications in languages other than English, potentially excluding the viewpoints and solutions to these challenges found within other cultures. Also, our review would not have captured all examples of implicit age-related bias, only in papers that mentioned the keyword “bias.” However, the studies included in this review can serve as exemplars of implicit and explicit bias. The implicit nature of ageism in the context of the technology sector with limited ethical oversight and regulations underscores the importance of further research and policy development. Our review also only examined papers that attempted to mitigate age-related bias, not other types of bias (such as gender- or ethnicity-related biases). One major concern is that these biases and omissions may continue to produce exclusion and push older people whose experiences are not read as “youthful” further outside the peripheries of shared social and cultural everyday spaces, including (but not limited to) those mediated through technological systems. This is perhaps especially the case where age intersects with locations already known to experience bias, such as gender, race, and ethnicity.

### Conclusion

This study explored and synthesized mitigation strategies for age-related bias in ML. The results underscore the value of 3 primary strategies for bias mitigation: data set balancing, data augmentation, and statistical techniques. However, the efficacy of these tactics demonstrated variability contingent upon factors such as data type, ML methodologies, and the intended purpose of the ML model. Although some researchers reported successful outcomes by diversifying their data sets, achieving complete elimination of bias remains challenging, and alternative approaches should be explored. The practical significance of the intervention should also be considered, as improvements in bias reduction may not always have substantial real-world impact, and further bias reduction and mitigation may still be required. A greater understanding of how digital ageism and age-related bias are propagated in ML and reproduced is needed across multiple sectors, including researchers and policy makers. Future research and policy agendas should include developing collaborative, comprehensive transdisciplinary strategies to foster fairness and inclusivity in the digital landscape.

## Appendix

Multimedia Appendix 1 Extracted information. The following data sets appeared in publications included in this review, but subject demographic data was not readily available; thus, they were not included in the tables: 11k Hands, ABIDE, CoRR, DLBS, NKI Rockland, Pilots Parliament Benchmark, and a data set comprised of Twitter Profile data.

Multimedia Appendix 2 PRISMA Checklist.

## Abbreviations

AAM: active appearance model
CAAE: Conditional Adversarial Auto Encoder
CACD: Cross-Age Celebrities data set
FACS: facial action coding system
FAN: facial alignment network
FER: facial emotional recognition
GAN: generative adversarial network
IPCGAN: Identity-Preserved Conditional Generative Adversarial Network
MAE: mean absolute error
ML: machine learning
MRI: magnetic resonance imaging
NRMSE: normalized root-mean square error
PACSLAC-II: Pain Assessment Checklist for Seniors with Limited Ability to Communicate-II
PRISMA-ScR: Preferred Reporting for Items for Systematic Reviews and Meta-Analyses Extension for Scoping Reviews

## Data Availability

Data sharing is not applicable to this article as no data sets were generated or analyzed during this study.
