# Supplementary material for: Strategies to Mitigate Age-Related Bias in Machine Learning: Scoping Review
Source: JMIR Aging. 2024 Mar 22;7:e53564. doi: 10.2196/53564 (PMC10998175; doi:10.2196/53564)
Supplement: Multimedia Appendix 2 [file aging_v7i1e53564_app2.pdf]

Preferred Reporting Items for Systematic reviews and Meta-Analyses extension for Scoping Reviews (PRISMA-ScR) Checklist

SECTION	ITEM	PRISMA-ScR CHECKLIST ITEM	REPORTED ON PAGE #
**TITLE**			
Title	1	Identify the report as a scoping review.	Page 1: "Strategies to Mitigate Age-related Bias in Machine Learning: A Scoping Review"
**ABSTRACT**			
Structured summary	2	Provide a structured summary that includes (as applicable): background, objectives, eligibility criteria, sources of evidence, charting methods, results, and conclusions that relate to the review questions and objectives.	Page 1 contains a structured abstract
**INTRODUCTION**			
Rationale	3	Describe the rationale for the review in the context of what is already known. Explain why the review questions/objectives lend themselves to a scoping review approach.	Page 4: "The topic of digital ageism is gaining prominence in scholarly discussions, leading researchers to investigate these phenomena from various perspectives [19,20,24,25]. Prior investigations have focused on developing conceptual frameworks to comprehend and define the nature and implications of digital ageism [13]. Previous reviews of facial-image datasets have also found that older adults, particularly older adults from the 85+ demographic, are under-represented in a majority of datasets [18]. While this research has been foundational in identifying and characterizing these biases, there is now a critical need to focus on the mitigation strategies that can address age-related bias in AI systems. The purpose of this scoping review is to advance this crucial discussion by shedding light on the mitigation strategies currently being used to address age-related bias in AI. By bridging the gap between theory and practice, this research aims to pave the way for meaningful and impactful interventions that can rectify biases and promote inclusivity in the digital age"
Objectives	4	Provide an explicit statement of the questions and objectives being addressed with reference to their key elements (e.g., population or participants, concepts, and context) or other relevant key elements used to	The research questions for this review can be found on page 4:<br>"1) Which mitigation strategies have been employed to address age-related bias in artificial intelligence, and how successful were these strategies?<br>2) Specifically, what types of biases

St. Michael's
Inspired Care.
Inspiring Science.

SECTION	ITEM	PRISMA-ScR CHECKLIST ITEM	REPORTED ON PAGE #
		conceptualize the review questions and/or objectives.	were targeted and mitigated during these efforts?”
**METHODS**			
Protocol and registration	5	Indicate whether a review protocol exists; state if and where it can be accessed (e.g., a Web address); and if available, provide registration information, including the registration number.	Page 5:<br>“The description of the review was published elsewhere and registered in the Open Science Framework database, under the doi: 10.17605/OSF.IO/AMG5P [26].”<br><br>The citation for the protocol can be found in the reference list on Page 28:<br>“26. Chu CH, Leslie K, Shi J, Nyrup R, Bianchi A, Khan SS, Rahimi SA, Lyn A, Grenier A. Ageism and Artificial Intelligence: Protocol for a Scoping Review. JMIR Res Protoc 2022 Jun 9;11(6): e33211. PMID:35679118”
Eligibility criteria	6	Specify characteristics of the sources of evidence used as eligibility criteria (e.g., years considered, language, and publication status), and provide a rationale.	Pages 5/6:<br>“Articles were included if they were published in English and focused on “artificial intelligence” in the context of algorithms that make predictions and classifications about data, “bias,” and age-related terms such as “aging,” “older,” and “demographic”. As the term “artificial intelligence” is over 50 years old [30], the search strategy was also not restricted by publication date and databases were searched from inception. Papers were excluded if they included non-human topics. Theses, conference abstracts, dissertations, non-peer-reviewed conference proceedings, books and book chapters, perspectives, editorials, and editorial letters were also excluded.”
Information sources*	7	Describe all information sources in the search (e.g., databases with dates of coverage and contact with authors to identify additional sources), as well as the date the most recent search was executed.	Page 5:<br>“Information Sources/Search<br>An information specialist helped develop the search strategy in Scopus, which was then translated into five other databases (Web of Science, CINAHL, EMBASE, IEEE Xplore, and ACM digital library). The search strategy included the terms: ‘machine learning’, ‘artificial intelligence’, ‘algorithms’, ‘neural networks’, ‘deep learning’, ‘algorithmic bias’; ‘biased’, ‘discrimination’, ‘ageism’; ‘age’, and ‘older people’.”<br><br>Page 6:

SECTION	ITEM	PRISMA-ScR CHECKLIST ITEM	REPORTED ON PAGE #
			“Selection of Sources of Evidence and Charting the Data<br>The academic literature search was completed in January 2022. All citations were uploaded to Covidence systematic review software (Veritas Health Innovation, Melbourne, Australia) and duplicates were removed.”
Search	8	Present the full electronic search strategy for at least 1 database, including any limits used, such that it could be repeated.	The following is the search strategy for the ACM Digital Library Database:<br><br>[[Publication Title: "artificial intelligence"] OR [Publication Title: "machine learning"] OR [Publication Title: "deep learning"] OR [Publication Title: "neural network*"] OR [Publication Title: algorithm*] OR [Abstract: "artificial intelligence"] OR [Abstract: "machine learning"] OR [Abstract: "deep learning"] OR [Abstract: "neural network*"] OR [Abstract: algorithm*] OR [Keywords: "artificial intelligence"] OR [Keywords: "machine learning"] OR [Keywords: "deep learning"] OR [Keywords: "neural network*"] OR [Keywords: algorithm*]] AND [[Publication Title: bias*] OR [Publication Title: discriminat*] OR [Publication Title: prejudice] OR [Publication Title: stigma] OR [Publication Title: stereotype*] OR [Abstract: bias*] OR [Abstract: discriminat*] OR [Abstract: prejudice] OR [Abstract: stigma] OR [Abstract: stereotype*] OR [Keywords: bias*] OR [Keywords: discriminat*] OR [Keywords: prejudice] OR [Keywords: stigma] OR [Keywords: stereotype*]] AND [[Publication Title: agis?] OR [Publication Title: ageis? or] OR [Publication Title: "old* people"] OR [Publication Title: "old* person*"] OR [Publication Title: "old* worker*"] OR [Publication Title: "old* adult*"] OR [Publication Title: "old* employee*"] OR [Publication Title: or senior*] OR [Publication Title: elder*] OR [Publication Title: age] OR [Publication Title: aging] OR [Publication Title: ageing] OR [Abstract: agis?] OR [Abstract: ageis? or] OR [Abstract: "old* people"] OR [Abstract: "old* person*"] OR [Abstract: "old* worker*"] OR [Abstract: "old* adult*"] OR [Abstract: "old* employee*"] OR [Abstract: or senior*] OR [Abstract: elder*] OR

SECTION	ITEM	PRISMA-ScR CHECKLIST ITEM	REPORTED ON PAGE #
			[Abstract: age] OR [Abstract: aging] OR [Abstract: ageing] OR [Keywords: agis?] OR [Keywords: ageis? or] OR [Keywords: "old* people"] OR [Keywords: "old* person*"] OR [Keywords: "old* worker*"] OR [Keywords: "old* adult*"] OR [Keywords: "old* employee*"] OR [Keywords: or senior*] OR [Keywords: elder*] OR [Keywords: age] OR [Keywords: aging] OR [Keywords: ageing]]
Selection of sources of evidence†	9	State the process for selecting sources of evidence (i.e., screening and eligibility) included in the scoping review.	Page 6, Section: "Selection of Sources of Evidence and Charting the Data"
Data charting process‡	10	Describe the methods of charting data from the included sources of evidence (e.g., calibrated forms or forms that have been tested by the team before their use, and whether data charting was done independently or in duplicate) and any processes for obtaining and confirming data from investigators.	Pages 6/7: Studies were selected if they acknowledged the presence of any bias against older adults in either their data or results and the researchers then took any action to correct that bias, regardless of its effectiveness. For example, publications were selected based on whether authors attempted to enhance the performance of their model on older demographics, regardless of the success of their efforts. Biweekly meetings were held to discuss progress of the charting process. Disagreements were resolved via discussion or by having the first author (CC) act as a third reviewer. The extracted information was converted into table format which allowed the authors to develop a narrative description according to the type of mitigation strategy (Table S1 of the Multimedia Appendix). The team conducted additional analysis of the databases in the included studies to identify data disparities of older adults and further directions for future studies in the field of AI and ageism. Results of the literature search are reported in tables. One of the challenges involved in assessing the inclusion of older adults involves defining the age at which someone is considered "old." While the commonly-accepted age for legal recognition as a "senior citizen," based on general eligibility for a public pension, is 65 years [31], the datasets and articles we reviewed grouped older adults into a much wider range of age categories (Table S2 of Multimedia Appendix)."

SECTION	ITEM	PRISMA-ScR CHECKLIST ITEM	REPORTED ON PAGE #
Data items	11	List and define all variables for which data were sought and any assumptions and simplifications made.	Datasets were analyzed for the age categories used to categorize their subjects, as well as the number of subjects in each category.<br><br>Publications were analyzed according to the framework for bias in Machine Learning, created by Mehrabi et al. Papers were included if they contained significant discussion of a specific type of bias, or whether or not they demonstrated said bias in their methods (for example, using a biased dataset would expose a paper's methods to representation bias).<br><br>Papers were included if their authors a) acknowledged that a certain type of bias against older adults was present in their paper, and then b) made a conscious effort to mitigate that bias. The effort did not need to be successful for the paper to be included, the authors only needed to make the attempt. For example, in Liang et a., the first effort at mitigating bias by balancing the dataset was unsuccessful, but the effort made by altering the algorithm was successful.
Critical appraisal of individual sources of evidence§	12	If done, provide a rationale for conducting a critical appraisal of included sources of evidence; describe the methods used and how this information was used in any data synthesis (if appropriate).	Papers were critically appraised for strategies used to mitigate bias against older adults specifically. Simply acknowledging that a bias affecting older adults was present in the data or outcomes of their publication was not enough, researchers had to attempt to mitigate it in some way.
Synthesis of results	13	Describe the methods of handling and summarizing the data that were charted.	Page 6, Section: "Selection of Sources of Evidence and Charting the Data"
**RESULTS**			
Selection of sources of evidence	14	Give numbers of sources of evidence screened, assessed for eligibility, and included in the review, with reasons for exclusions at each stage, ideally using a flow diagram.	See Figure 1 for a PRISMA flowsheet of the results.
Characteristics of sources of evidence	15	For each source of evidence, present characteristics for which data were charted and provide the citations.	See Table 1 in the Multimedia Appendix
Critical appraisal within sources of	16	If done, present data on critical appraisal of included sources of evidence (see item 12).	See Table 2 in the Multimedia Appendix for a critical appraisal of common image datasets.

SECTION	ITEM	PRISMA-ScR CHECKLIST ITEM	REPORTED ON PAGE #
evidence			Page 8: Datasets Used in the Included Studies
Results of individual sources of evidence	17	For each included source of evidence, present the relevant data that were charted that relate to the review questions and objectives.	See Tables 1 and 2 in the Multimedia Appendix<br><br>Page 9: Bias Mitigation Strategies
Synthesis of results	18	Summarize and/or present the charting results as they relate to the review questions and objectives.	Page 8: Representation bias affecting older adults was present in the most commonly-used datasets in the study (Table 2 in the Multimedia Appendix).<br><br>Pages 9-17: The three strategies which emerged in our work included dataset balancing, dataset augmentation, and algorithmic alteration (Table 1 in the Multimedia Appendix), with one paper attempting algorithmic alteration after the initial attempt at dataset balancing proved unsuccessful.
**DISCUSSION**			
Summary of evidence	19	Summarize the main results (including an overview of concepts, themes, and types of evidence available), link to the review questions and objectives, and consider the relevance to key groups.	Pages 18-23: Discussion
Limitations	20	Discuss the limitations of the scoping review process.	Pages 23-24: Limitations
Conclusions	21	Provide a general interpretation of the results with respect to the review questions and objectives, as well as potential implications and/or next steps.	Page 24: Conclusion
**FUNDING**			
Funding	22	Describe sources of funding for the included sources of evidence, as well as sources of funding for the scoping review. Describe the role of the funders of the scoping review.	Project funded by the Social Sciences & Humanities Research Council (SSHRC) of Canada

JBI = Joanna Briggs Institute; PRISMA-ScR = Preferred Reporting Items for Systematic reviews and Meta-Analyses extension for Scoping Reviews.

* Where *sources of evidence* (see second footnote) are compiled from, such as bibliographic databases, social media platforms, and Web sites.

† A more inclusive/heterogeneous term used to account for the different types of evidence or data sources (e.g., quantitative and/or qualitative research, expert opinion, and policy documents) that may be eligible in a scoping review as opposed to only studies. This is not to be confused with *information sources* (see first footnote).

‡ The frameworks by Arksey and O'Malley (6) and Levac and colleagues (7) and the JBI guidance (4, 5) refer to the process of data extraction in a scoping review as data charting.

§ The process of systematically examining research evidence to assess its validity, results, and relevance before using it to inform a decision. This term is used for items 12 and 19 instead of "risk of bias" (which is more applicable

to systematic reviews of interventions) to include and acknowledge the various sources of evidence that may be used in a scoping review (e.g., quantitative and/or qualitative research, expert opinion, and policy document).

From: Tricco AC, Lillie E, Zarin W, O'Brien KK, Colquhoun H, Levac D, et al. PRISMA Extension for Scoping Reviews (PRISMAScR): Checklist and Explanation. Ann Intern Med. 2018;169:467–473. doi: 10.7326/M18-0850.
